# Upscaling Transport of *Bacillus subtilis* Endospores and Coliphage phiX174 in Heterogeneous Porous Media from the Column to the Field Scale

**DOI:** 10.1021/acs.est.1c01892

**Published:** 2021-07-28

**Authors:** Thomas J. Oudega, Gerhard Lindner, Julia Derx, Andreas H. Farnleitner, Regina Sommer, Alfred P. Blaschke, Margaret E. Stevenson

**Affiliations:** † Institute of Hydraulic Engineering and Water Resources Management E222/2, TU Wien, Karlsplatz 13, A-1040 Vienna, Austria; ‡ Research Group Environmental Microbiology and Molecular Diagnostics 166/5/3, Institute of Chemical, Environmental and Bioscience Engineering, TU Wien, Gumpendorferstraße 1a, A-1060 Vienna, Austria; ∥ Medical University of Vienna, Institute for Hygiene and Applied Immunology, Water Hygiene, Kinderspitalgasse 15, A-1090 Vienna, Austria; ⊥ Karl Landsteiner University for Health Sciences, Department Physiology, Pharmacology and Microbiology, Research Division Water Quality & Health, 3500 Krems, Austria; # Interuniversity Cooperation Centre (ICC) Water & Health, A-1060 Vienna, Austria

**Keywords:** microbial tracer tests, upscaling column to field, 3D colloidal transport modeling

## Abstract

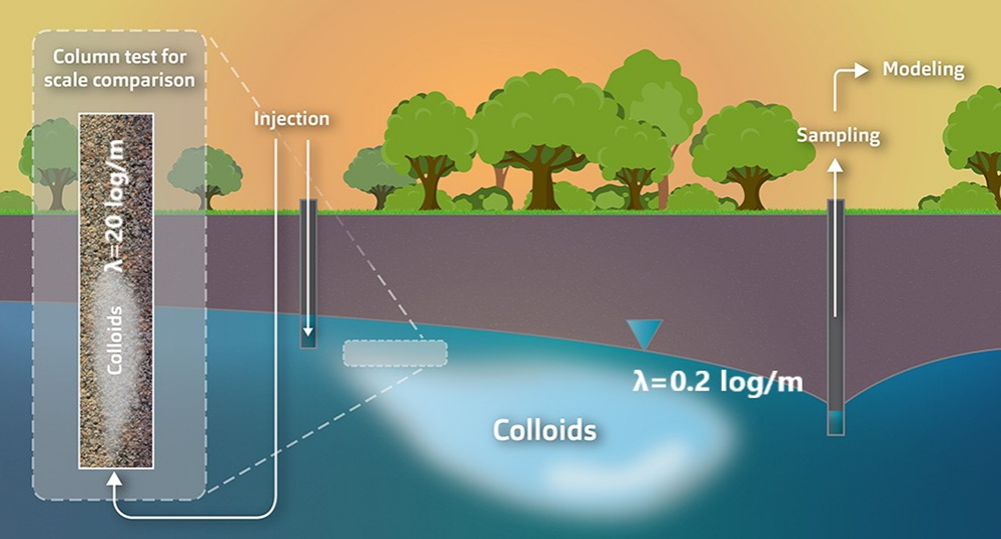

Groundwater contamination and transport of viruses and bacteria in aquifers are a major concern worldwide. To ascertain the ability of these aquifers to remove pathogens, tracer tests with microbial surrogates are carried out. These tests are laborious and may require special permits, and therefore, column tests are often done instead. Unfortunately, results from column tests tend to grossly overestimate removal rates when compared to the field scale, which can lead to an underestimation of groundwater contamination risks. Scale is an important consideration when examining pathogen transport through porous media, as pathogen removal is rarely a linear process. In this study, field tests were carried out with endospores of *Bacillus subtilis* and coliphage phiX174 over a distance of 25 m in an alluvial gravel aquifer near Vienna, Austria. The sandy gravel material from the field site was also used in column tests with the same tracers. Both attachment-detachment and colloid filtration theory were used to model these tests, as well as log-removal rates per meter. The results show that the spatial removal rate (log/m) is approximately 2 orders of magnitude higher on the column scale, when compared to the field. A comparison with the literature showed a correlation between the heterogeneity of the porous media and the difference in removal rates between the column and field scale.

## Abstract 2

Tracer tests at different scales showed that preferential flow is an important factor affecting upscaling of microbial transport.

## 1 Introduction

Groundwater is an important source of drinking water for many people around the world. Disease outbreaks due to contaminated groundwater are, therefore, of great concern.^1−3^ In the last few decades, multiple disease outbreaks across the U.S. and Europe have been shown to have had their origin in contaminated groundwater, but it is difficult to identify specific risks due to a substantial lack of data.^4−6^ It can be assumed that many cases of water-associated infections go undocumented in developing countries as well as in affluent nations.^7^ Furthermore, while health risks associated with surface water contamination have been decreasing since the end of last century, this is not the case for groundwater.^8^

One economically advantageous treatment option to reduce the concentration of pathogens in groundwater is to ensure sufficient transport times through the porous media in question. In order to determine the pathogen removal rates in an aquifer, tracer tests with surrogate organisms are often performed;^9,10^ however, field tracer tests may require special permits and are time-consuming, expensive, and site-specific. Removal of pathogens in the subsurface varies greatly depending on the type of microorganism and its interaction with site-specific aquifer material, so it is often impossible to transfer the results from one site to another.^11^ Soil characteristics, such as chemical attributes, rock fractures, lenses of higher permeability, and physical heterogeneity, can negatively influence the removal of pathogens during transport.^12−14^ Though it has been shown that preferential transport pathways are responsible for decreased pathogen removal rates in porous media, more research is needed to adequately describe these processes.^15^

Although it has been stated that more field tests are crucial for our understanding of removal processes, there is a severe lack of these tests at the field scale,^16^ and therefore, studies on microbial removal are often done in columns in the laboratory. Unfortunately, observed removal processes in columns might not be representative of the field scale, and this method often grossly overestimates microbial removal rates and parameters controlling attachment.^17,18^ Thus, it is essential to understand how scale affects colloidal transport in porous media and to identify the dominant factors that influence the upscaling of transport processes.

Most upscaling research focuses largely on theory, for example, by using different modeling approaches to upscale column results to the field scale. These include stream tube models that mimic preferential flow in the field by aggregating different one-dimensional flowpaths.^19−21^ This promising concept is, however, rarely coupled with field observations. In this study we aimed to compare tests done at the field and column scale using microorganisms of different sizes. The same aquifer material was used at both scales to ensure comparability. Using two different modeling methods (attachment-detachment theory and colloid filtration theory) as well as log-removal rates calculated per meter, these tests were compared to each other and the literature, in order to gain insight into removal processes at different scales. We hypothesize that one reason average removal rates per meter are higher at the column scale when compared to the field scale is because of soil heterogeneity and the fact that preferential flow elements, such as cracks or lenses of coarser material, are not captured at the column scale.

## 2 Materials and Methods

### 2.1 Field Study Site

Field tests were carried out at the Obere Lobau test site located near Vienna, Austria. The site consists of an injection well (P24) and a pumping well (LB13) at a distance of 25 m. The injection well has a diameter of 51 mm, a depth of 14 m, and the well screen is from 8 to 14 m below the ground surface. The pumping well has a depth of 24 m, a well screen from 5 to 23 m depth, and the pump is located at a depth of 21 m.

**Figure 1:**
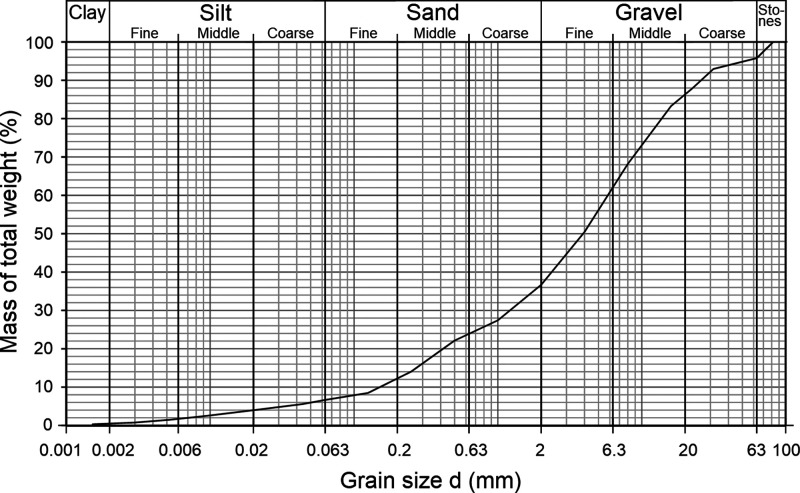
Sieve analysis of soil material taken from P24 from a depth of 11 to 12 m.

The study site is located next to the River Danube, but is separated from it by a dam with an impermeable core, and is therefore not under the influence of flooding from the river. The site consists of alluvial sediments including gravel, sand, and clay, overlain by an approximately 2 m deep sandy silt soil. Loose sandy and semiconsolidated gravels occur from 2 m to a maximum depth of 35 m. These gravel layers are interrupted and underlain by thin clayey silt and consolidated layers, intermittent throughout the area. While gravel accounts for the largest portion of the aquifer grain size distribution, a notable amount of fine material is present as well (Figure 1). The uniformity coefficient *C*_U_ (d_60_ d_10_^–1^), a measure of uniformity of the soil, equals 38.4 for our test site, and the coefficient of curvature, *C*_C_ (d_30_^2^ (d_60_·d_10_)^−1^), is 1.68. Soils are considered uniform if *C*_U_ < 4 and *C*_C_ is between 1 and 3 and low uniformity (*C*_U_ > 4) leads to higher dispersivity.^22,23^ The bulk density of the material is 2.24 g cm^–3^. The groundwater at the site is iron-rich and anoxic (Table 1).

**Table 1:** Chemical Analysis of Groundwater and Viennese Tap Water^a^

	groundwater properties	tap water properties
water level depth (m)	4.0–6.5	
groundwater gradient	0.0014	
pH	7.3	8.0
EC (μS cm^–1^)	637	266
temperature (°C)	10.5–11.2	8.6–10.0
oxygen (mg L^–1^)	0.0–0.6	9.7–10.3
TOC (mg L^–1^)	1.1	0.4
iron (mg L^–1^)	2.1	<0.05
manganese (mg L^–1^)	0.4	<0.02
chloride (mg L^–1^)	13	2.6
sodium (mg L^–1^)	9.1	1.2
calcium (mg L^–1^)	64	46
kalium	2.0	<0.5
magnesium	14	9.3
sulfate	21	11
nitrite (mg L^–1^)	<0.01	<0.01
nitrate (mg L^–1^)	<1	4.9

a Electrical Conductivity (EC), Total Organic Carbon (TOC).

### 2.2 Tracer Preparation and Analysis

Sodium bromide (NaBr) was used in each test as a conservative tracer. Restrictions in regards to the concentration of the injected tracers in the field were imposed by the authorities of the City of Vienna and the maximum concentration of NaBr to be injected was 100 mg L^–1^, and for microorganisms, 10^12^ PFU L^–1^ and CFU L^–1^, respectively. In the field, bromide samples were taken by an autosampler and stored in plastic test tubes. Analysis was performed at the TU Wien with HP/LC Chromatography (Metrohm ECO IC, Herisau, Switzerland), no longer than 2 days after sampling. During column tests, electrical conductivity (EC) measurements were carried out by means of a flow-through cell.

*Bacillus subtilis* is a rod-shaped, Gram-positive, aerobic, nonpathogenic bacterium present in low-temperature environments.^24^ Under nonfavorable conditions the vegetative bacterium is able to form endospores. The size of the spores was measured with an electron microscope (FEI Company, Hillsboro, U.S.A.) to be 1.5 μm in length and 0.5 μm in width. *B. subtilis* spores have an overall negative charge.^25^ The spores of strain ATCC 6633 were produced from freeze-dried form. The day before use, a defined amount of the freeze-dried powder was suspended at room temperature in deionized water, treated in an ultrasonic bath for about 5 min and thoroughly vortexed to break up any clumps in the suspension before being stored at 4 ^○^C. This suspension was injected within 3 days of preparation. Samples were stored in glass test tubes, cooled and brought to the Medical University of Vienna for analysis. Before processing, the samples were heat treated at 70 ^○^C for 10 min to inactivate vegetative cells, no later than 24 h after collection. Incubation was at 36 ± 2 ^○^C for 44 ± 4 h on plate count agar (Tryptone Glucose Yeast Extract, Oxoid, Hampshire, U.K.). The enumeration was done by counting the colonies formed.

PhiX174 is a single-stranded DNA, nonenveloped, virus with a size of 26 nm and a spherical shape.^26,27^ Its host cell is *Escherichia coli*, and it is not pathogenic for humans.^28^ Even though phiX174 has a close to zero charge at near-neutral pH,^28−30^ it is generally seen as a good viral surrogate for virus transport, because of its stability and low hydrophobicity.^31−33^ PhiX174 may not be an ideal conservative colloidal tracer, but somatic coliphages have gained special importance in Europe in recent years because of its usefulness as a reliable viral fecal indicator due to their high prevalence in sewage and their persistence in the environment. PhiX174 viruses were prepared following the international standard ISO 10705–2.^34^ The virus stock suspension was suspended in deionized water and injection was performed within 48 h of preparation. The enumeration was done by double layer semisolid agar overlay method. *Escherichia coli* of strain ATCC 700078 was used as a host and incubation was at 36 ± 2 °C for 18 ± 2 h. The volumes of processed samples were 3 to 9 mL, depending on the presumed concentrations of microorganisms.

The zeta potentials of both colloids were measured by electrophoretic light scattering (Malvern Pananalytical Zetasizer Nano ZSP, U.K.). The concentrations of microorganisms used for the measurements were the same as during injection (10^6^ CFU mL^–1^). The measurements were done in Lobau groundwater, Vienna tap water, and 10 mM NaCl buffered to both pH 7.3 and 8.0. All background matrices were sterile filtered before the measurements. The measurements were carried out in triplicate.

### 2.3 Field Experiments

Tracer experiments were done in duplicate with spores of *B. subtilis* and phage phiX174, injected separately. The groundwater gradient between P24 and LB13 was approximately 0.02 for all tests, and the difference in gradient between duplicate experiments was never greater than 2.8‰ (or 7 cm). The pumping rate of 5 L s^–1^ was kept constant for at least 72 h before injection to ensure a stable groundwater table. One hour before injection, a sample was taken to verify that there was no background concentration of *B. subtilis* or phiX174. A suspension of a microbial tracer was injected with a total amount of 1.65 × 10^12^ and 1.16 × 10^12^ CFU spores of *B. subtilis* in 1.5 l groundwater, for the first and second test, respectively; and 2.1 × 10^12^ and 2.3 × 10^12^ PFU phage phiX174 in 1.0 and 1.5 l groundwater, respectively. The suspension was injected in P24 by means of peristaltic pump, in 1 to 2 min. This was immediately followed by an injection of 100 g NaBr in a volume of 1000 L of groundwater by fuel pump, which took approximately 15 min. These injections were done below the water table (at a depth of 7 m) to minimize the amount of oxygen added to the groundwater. Injection did not affect the water levels in the surrounding piezometers during the tests.

### 2.4 Column Experiments

**Figure 2:**
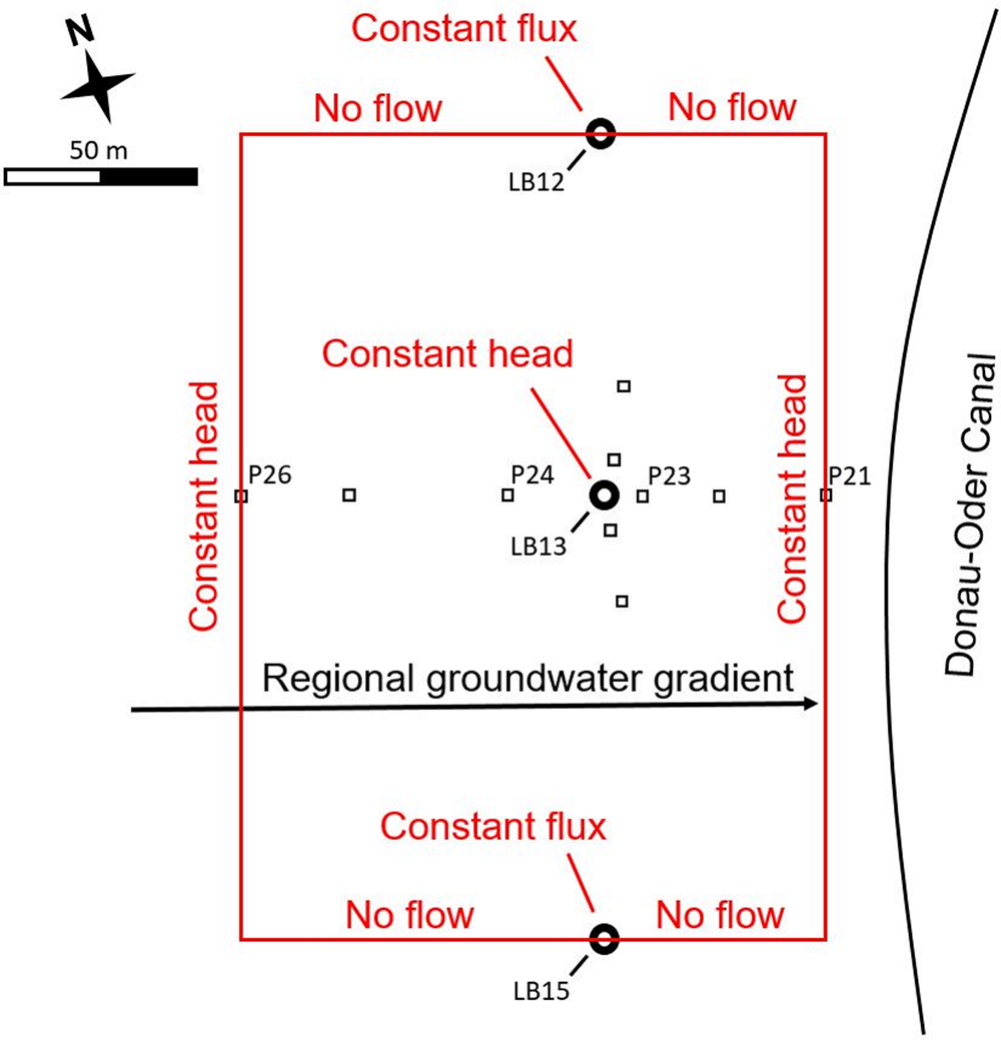
Overview of the field site with the model domain and boundary conditions in red. LB wells (circles) are pumping wells, P wells (squares) are piezometers.

The 500 mm long × 70 mm diameter Plexiglas column was freshly packed with new material from the field site for each column test. The material was taken at a depth of 11 to 12 m, either from P24 or from the closest well to the pumping well (*P*23, Figure 2). Stones larger than 5 cm were removed, as these would affect water flow too much in a column with a 70 mm diameter,^35^ and the ratio of *d*_col_/*d*_50_ (the inner column diameter divided by the effective grain size) was 350, much higher than the recommended minimum ratio of 50, to ensure minimal potential wall effects in the column.^36,37^

The influent water used in the column tests was standard Viennese tap water, which is Alpine karstic spring water with a pH of approximately 8.0 and an EC of 250 μS cm^–1^. In contrast to the groundwater of the field site, the tap water was oxic and low in iron (Table 1). The columns were rinsed for a minimum of 20 pore volumes (PV) and the experiments were run at a flow rate of approximately 18 mL min^–1^, which was the highest possible flow rate in the column without a buildup of pressure due to the fine material, causing the tubes to burst off at the connections. For each column test, the porosity and the flow rate were measured.

An 800 mL solution was injected, containing 400 mL with the colloidal tracers prepared separately for the four different tests (1.08 × 10^9^ and 4.00 × 10^9^ spores of *B. subtilis*; 2.24 × 10^8^ and 2.30 × 10^9^ phages phiX174) and 400 mL tap water with 2 mM L^–1^ NaBr. This was injected with a peristaltic pump from an automatically stirred Erlenmeyer flask. Duplicate tests were carried out for each tracer. Samples were taken by hand every 2 min, while the EC was measured in a flow-through cell between sampling intervals.

### 2.5 Field Test Modeling

The field tests were modeled using HYDRUS 2/3D software.^38^ The three-dimensional domain was defined as a cuboid with a depth of 24 m, as local drillings suggest that an aquiclude exists at this depth, comprising of a thick clay layer. The domain boundaries upstream and downstream were assigned constant heads at P26 and P21, respectively, the furthest points from the pumping well where reliable water level data was available for all tests (Figure 2). In the transverse direction, no-flow boundaries were located at the first pumping wells on either side of the flow line, at a distance of 105 m north (LB12) and 152 m south (LB15) of the pumping well, LB13. The pumping wells LB12 and LB15 were assigned constant flux boundary conditions of 7.2 l s^–1^ and 12.5 l s^–1^, respectively.

The well screen was defined as a cylinder with a diameter of 30 cm. This was modeled as a constant head boundary, in order to have full control over the gradient between the points of tracer injection and extraction. No flow boundary conditions were assigned to the top and bottom of the domain.

Parameter estimation of porosity and hydraulic conductivity in HYDRUS 2/3D was done by trial and error. Calibration was based on water level measurements and conservative tracer tests, carried out with bromide. These values were then used during the subsequent modeling of the microbial transport using the advection-dispersion equation, one-site attachment-detachment model and colloid filtration theory (CFT) equations (eqs S1–S3 of the Supporting Information, SI). Due to the fact that our field site has poorly sorted soils, instead of using the grain size *d*_50_ for the parameter d in CFT (eq S3, SI), *d*_10_ was used. This was considered to be a reasonable assumption because in soils with high amounts of fine material, this size fraction is more important in the removal of colloids than the median size fraction.^39−41^

### 2.6 Column Test Modeling

The column tests were modeled with the HYDRUS-1D software package.^42^ The boundary conditions were defined as a constant pressure head on both ends of the column. HYDRUS-1D uses a nonlinear least-squares optimization routine, which allows for the inverse estimation of parameters by fitting to observation data. This was used to find values for dispersivity based on the bromide BTC. These values were then used during the subsequent modeling of the microbial transport using the advection-dispersion equation (adapted for one dimension), the one-site attachment-detachment model and the CFT model, as per eqs S1–S3 (SI).

### 2.7 Data Analysis

Breakthrough curves of the microbial and conservative tracers were plotted over time and normalized to the initial concentration. From this data, spatial microbial removal rates (λ, log reduction L^–1^) were calculated for each test as per eq 1, which is valid for three dimensions if the flow is parallel to the *x*-direction:^43^
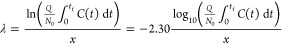
1where *Q* is equal to the flow rate [L^3^ T^–1^], *N*_0_ is the total amount of tracer injected [M], *C*(*t*) the concentration at a given time *t* after injection [M L^–3^], *t*_f_ is the final time of the test after the pulse has passed through [T], and *x* the distance from the injection point to the sampling point [L]. The integration was approximated by dividing the time series into sampling intervals, of which it was assumed that the sample concentration was an average value.

## 3 Results and Discussion

### 3.1 Zeta Potentials

The zeta potentials for both microbes were measured in 4 different sterile filtered matrices: groundwater sampled from P24 at the field site, Vienna tap water used for the column tests and a standard 10 mM NaCl solution buffered to pH 7.3 and 8.0 using NaHCO_3_, for literature comparison. The zeta potentials of spores of *B. subtilis* in Lobau groundwater, Vienna tap water, and NaCl buffer pH 7.3 and 8.0, were −17.65 ± 0.05, −18.40 ± 1.02, −47.40 ± 0.92, and −30.85 ± 0.45 mV, respectively. For phiX174 the values were −18.47 ± 0.23, −5.15 ± 0.63, −3.27 ± 0.46, and −4.57 ± 2.23 mV. The values measured for spores of *B. subtilis* were comparable to other studies, in which values of −8 to −15 mV in pH 7, deionized water,^44^ and −31.5 mV in pH 6.92, groundwater^40^ were found. In contrast, zeta potentials of −19 to −10 mV were found in a 10 mM pH 7.5 NaCl solution,^45^ which is less negative than in our study. This might be influenced by the strain of *B. subtilis* used (ATCC7058 and ATCC15811). The zeta potentials of phiX174 were also similar to values in the literature such as −7.5 mV in pH 7 biologically filtered water,^46^ and −8.3 mV in pH 7.3, 154 mM NaCl.^27^ In double-distilled water (ddH_2_O) at pH 7, the zeta potential of phiX174 was −31.78 mV, and it became less negative as the pH increased after reaching a minimum around a pH of 5.5.^47^ This may explain why phage phiX174 was less negative in Vienna tap water, which had a higher pH than the Lobau groundwater, whereas the zeta potentials of spores of *B. subtilis* were similar in Vienna tap water and Lobau groundwater.

### 3.2 Field Test Results

**Figure 3:**
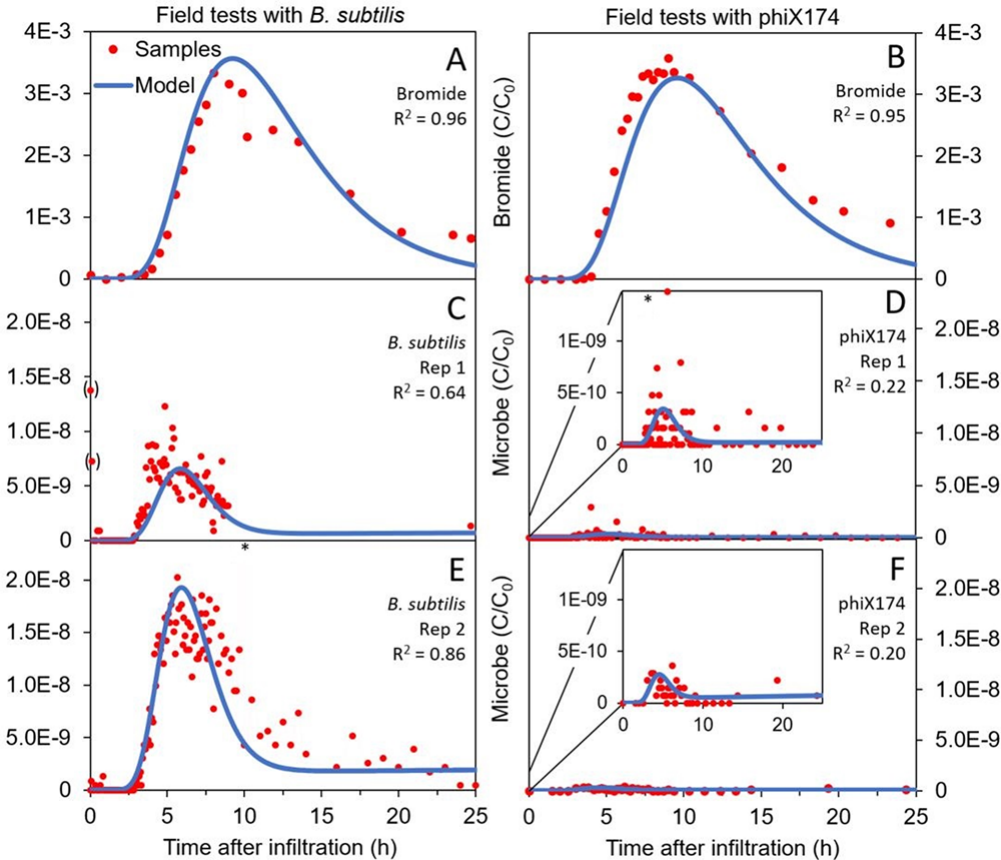
Measured samples (red) and modeling results (blue) for the BTCs in field tests with bromide and replicate field tests with spores of *B. subtilis* and phage phiX174 (*C*/*C*_0_). Samples interpreted as contamination are represented as a red point between brackets. The asterisk (*) stands for samples with a concentration higher than the maximum on the *y*-axis.

The transport and retention behavior of spores of *B. subtilis* and phage phiX174 at the field site is shown in Figure 3, in which the breakthrough of both microorganisms precedes that of bromide. This is most probably due to pore size exclusion, which traps smaller colloids and dissolved compounds in narrow pore spaces, allowing the center of mass of the pulse of larger colloids to move faster.^48^ Generally, colloidal detachment in all tests was observed to be low. Both breakthrough curves (BTCs) of bromide (Figure 3A,B) peaked approximately 8 h after injection. The peak breakthrough concentration of spores of *B. subtilis*, around 5 h after injection, was approximately 2 orders of magnitude higher than that of phiX174, which also peaked 4 to 5 h after injection. Similarly, the percentage of mass recovery of spores of *B. subtilis* was 100 times greater than for phage phiX174 (Table 2).

**Table 2:** Comparison of Modeling Results between the Microbial Tracers and Column/Field Scale^a^

field tests	*B. sub.* 1	*B. sub.* 2	PhiX174 1	PhiX174 2
flow rate (m h^–1^)**	0.18–3.43	0.15–3.12	0.16–3.00	0.17–3.36
peak breakthrough (*C*/*C*_0_)	1.09 × 10^–8^	2.03 × 10^–8^	2.91 × 10^–8^	3.62 × 10^–10^
microbial mass recovered (%)	0.1085	0.1645	0.0039	0.0031
first-order removal rate λ (log m^-1^)*	0.23	0.21	0.34	0.35
longitudinal dispersivity *D*_x_ (m)*	1.2	1.2	1.5	1.5
transverse dispersivity *D*_y_ (m)*	0.15	0.15	0.15	0.15
porosity θ (−)**	0.12	0.12	0.12	0.12
attachment rate *K*_att_ (h^-1^)*	0.95	1.01	1.33	1.73
detachment rate *K*_det_ (h^-1^)*	4.0 × 10^–3^	3.0 × 10^–3^	2.0 × 10^–3^	8.5 × 10^–3^
*K*_att_/*K*_det_(−)	3.4 × 10^2^	3.4 × 10^2^	6.4 × 10^2^	2.0 × 10^2^
collision efficiency η (−)	5.70 × 10^–2^	5.87 × 10^–2^	2.11 × 10^–1^	2.00 × 10^–1^
attachment efficiency α (−)*	5.95 × 10^–4^	4.09 × 10^–4^	1.61 × 10^–4^	2.03 × 10^–4^
removal efficiency α · η (−)	3.39 × 10^–5^	2.40 × 10^–5^	3.40 × 10^–5^	4.05 × 10^–5^
coefficient of determination (*R*^2^)	0.644	0.859	0.222	0.199

column tests	*B. sub.* 1	*B. sub.* 2	PhiX174 1	PhiX174 2
flow rate (m h^-1^)	0.28	0.28	0.23	0.26
peak breakthrough (*C*/*C*_0_)	1.09 × 10^–8^	5.07 × 10^–6^	8.93 × 10^–6^	7.23 × 10^–5^
microbial mass recovered (%)	0.0093	0.0041	0.0014	0.0073
first-order removal rate λ (log m^-1^)	18.55	20.20	22.31	19.04
dispersivity *D*_x_ (cm)*	1.91 ± 1.46	2.27 ± 4.54	1.24 ± 0.21	2.48 ± 0.13
porosity θ (−)	0.187	0.171	0.197	0.184
attachment rate *K*_att_ (h^-1^)*	50.77 ± 2.00	117.82 ± 5.07	36.56 ± 1.48	40.09 ± 0.70
detachment rate *K*_det_ (h^-1^)*	4.8 × 10^–2^ ± 2.9 × 10^–2^	5.9 × 10^–2^ ± 2.3 × 10^–2^	7.55 × 10^–3^ ± 2.77 × 10^–3^	9.16 × 10^–3^ ± 3.18 × 10^–3^
*K*_att_/*K*_det_(−)	1.06 × 10^3^ ± 6.4 × 10^2^	2.00 × 10^3^ ± 7.8 × 10^2^	4.84 × 10^3^ ± 3.1 × 10^3^	4.38 × 10^3^ ± 2.1 × 10^3^
collision efficiency η (−)	3.99 × 10^–2^	3.98 × 10^–2^	3.16 × 10^–1^	2.92 × 10^–1^
attachment efficiency α (−)*	0.1014 ± 0.0029	0.1861 ± 0.0048	0.0113 ± 6.5 × 10^–5^	0.0107 ± 1.1 × 10^–4^
removal efficiency α · η (−)	4.05 × 10^–3^ ± 1.20 × 10^–4^	7.41 × 10^–3^ ± 1.90 × 10^–4^	3.57 × 10^–3^ ± 2.10 × 10^–5^	3.12 × 10^–3^ ± 3.20 × 10^–5^
coefficient of determination (*R*^2^)	0.040	0.007	0.785	0.934

a Standard deviations are given for parameters that were fitted by inverse optimization for column test models. *fitted to the microbial breakthrough curve. **fitted to the bromide breakthrough curve.

### 3.3 Field Test Modeling

A hydraulic conductivity of 7.5 × 10^–3^ m s^–1^ and a total porosity of 0.12 were calibrated on tests with bromide by trial and error in the one layer model, and are considered realistic for very heterogeneous, coarse gravel.^49^ A longitudinal and transverse dispersivity of 1.8 and 0.18 m, respectively, were found to simulate the BTC of the bromide best. The values for porosity and hydraulic conductivity were kept the same for the modeling of spores of *B. subtilis* and phage phiX174. The BTCs of microbial tracers were earlier and less dispersed than the BTCs of bromide. During model calibration a lower dispersivity value for the microorganisms was found (Table 2), which indicates the presence of pore size exclusion in our tests.^40,50^ The coefficients of determination (*R*^2^) are low for the modeling of phiX174, which is most likely because of the low breakthrough concentrations (Figure 3D,F).

Inactivation of *B. subtilis* was not modeled because it is usually insignificant in saturated column studies, as well as field studies.^28,51−53^ The inactivation rate of phiX174 was found to be about 1-log over a course of 12 weeks in groundwater in a gravel aquifer by DeBorde et al., and was therefore assumed to be negligible on the time scale of this study (1 day).^54^ Straining was not considered because for both microbes, the fraction of colloid size to median grain size was lower than 0.017, and therefore straining should not occur.^55^ Wedging may occur at smaller colloid/grain ratios and could be an issue for the spores in our study.^56^ Additionally, natural sand is angular and known to lead to a higher collision efficiency (η) than spherical grains.^57^ For these reasons, removal efficiencies (η·α) are reported, representing both the colloidal collision and the resulting attachment, in order to compare the attachment of phiX174 and *B. subtilis* in the context of CFT.

In the modeled field tests, phage phiX174 exhibited a higher attachment rate *K*_att_ (h^–1^) than spores of *B. subtilis* (Table 2). The detachment rates *K*_det_ (h^–1^) were similar between the two microbial tracers. According to CFT, attachment efficiencies (α) were 2 to 3 times higher for spores of *B. subtilis* than for phage phiX174, even though their zeta potential were similar in Lobau groundwater. Collision efficiencies (η), however, were around 4 times higher for phiX174, owing to Brownian motion due to their much smaller size.^58^ This led to slightly higher removal efficiencies (α·η) for phiX174 in our models. A higher removal of viruses compared to bacteria in heterogeneous aquifer material was also by observed other authors.^59,35^ An explanation for this phenomenon might be that PhiX174 in particular is not as conservative as *B. subtilis*, due to its zeta potential being less negative under some conditions. Alternatively, larger colloids are more likely to be transported quickly through lenses of course material, resulting in less attachment, and smaller colloids are partially dispersed in dead end narrow pore zones, into which the larger colloids cannot enter.^16,60,61^

### 3.4 Column Test Results

**Figure 4:**
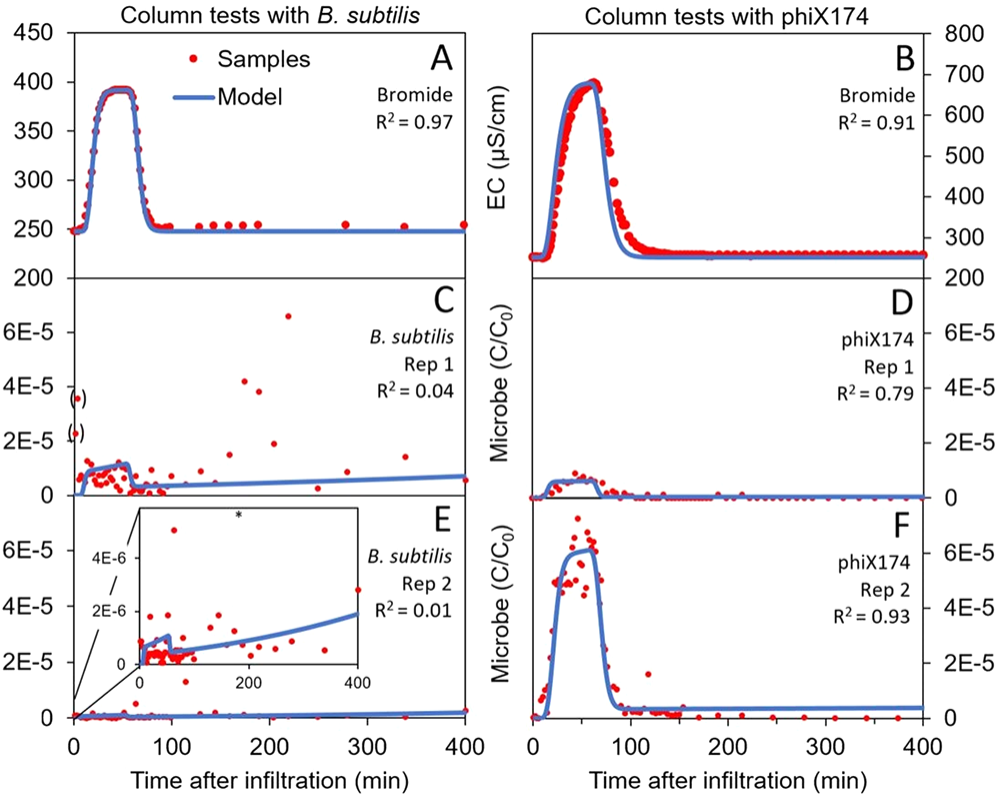
Measured samples (red) and modeling results (blue) for the BTCs in column tests with bromide (μS cm^–1^) and replicate column tests with spores of *B. subtilis* and phage phiX174 (*C*/*C*_0_). Samples interpreted as contamination are represented as a red point between brackets. The asterisk (*) stands for samples with a concentration higher than the maximum on the *y*-axis.

In contrast to the field test results, the breakthrough of bromide and phage phiX174 were approximately at the same time in the column tests (Figure 4B,D,F). Surprisingly, the breakthrough of the spores of *B. subtilis* was much earlier, even arriving in the first samples taken (Figure 4A,C,E).

As seen in the field tests, removal rates in the columns were lower for *B. subtilis* compared to phiX174 (Table 2), leading to a higher mass recovery. In both column tests with *B. subtilis*, there was high detachment after the initial peak concentration. Unfortunately, this could not be modeled correctly because the detachment concentrations were too erratic, which led to very low coefficients of determination (R^2^) for *B. subtilis* (Figure 4C, E).

### 3.5 Column test modeling

Using Hydrus-1D, it was found that both K_att_ and K_det_ were higher for *B. subtilis*, compared to phiX174. Attachment efficiencies (α) were around 1 order of magnitude higher for *B. subtilis* than for phiX174. Even so, the removal efficiencies (α·η) were similar for both microbes, because of a higher collision efficiency (η) of phiX174. This led to a similar recovery of *B. subtilis* and phiX174 (Table 2). The high and irregular detachment of *B. subtilis* led to high concentrations in the outflow, even after the initial peak had passed. This was not observed in the tests with phiX174, which might be explained by the difference in zeta potential; phiX174 was less negative in Vienna tap water than *B. subtilis*. More negatively charged colloids generally have a higher breakthrough due to higher electrostatic repulsion with negatively charged porous media.^62^ In Lobau groundwater, the two microbes have similar zeta potentials. It may be that, for this reason, detachment looks similar in the field BTCs for both microbes.

### 3.6 Upscaling of Modeled Parameters

The removal rate (λ) for *B. subtilis* in our field study was higher than found by others in similar soils.^17,63^ The gravel material from our field site has a high amount of fine material, which might be a reason for this discrepancy.^55,64^ Alternatively, the different strains used in the other studies (e.g., strain JH1) might be why our λ was higher, for example because our strain had a weaker negative charge (−18 mV versus −31 mV of strain JH1, both measured in groundwater).^65^ In contrast, removal rates (λ) in this study were similar to those found in tests done with other bacteria of a similar size, such as *E. coli*.^9,17^ For phiX174, we found similar λ, *K*_att_, and α values, compared to other studies with phiX174 or similar bacteriophages (such as *PRD1* or *MS2*), both on the field and column scale.^9,18,36,59,66,67^

As no more can detach than has attached, detachment rates (*K*_det_) are always lower than attachment rates (*K*_att_) at all scales and, therefore, the ratio of *K*_att_/*K*_det_ cannot be less than 1. In our study we looked at this ratio (*K*_att_/*K*_det_) at the column scale and compared it to the same ratio at the field scale, as a way to evaluate upscaling effects (Table 2). It has been hypothesized that this ratio may be the same at all scales;^68^ however, in our study, the ratio *K*_att_/*K*_det_ for both colloidal tracers was higher for the column tests than for the field tests by about 1 order of magnitude. Thus, a stable *K*_att_/*K*_det_ ratio was not found between the column and field scale. As it was difficult to model the falling limb of the BTCs, due to the erratic nature of the detachment, we could not verify with certainty if the ratio *K*_att_/*K*_det_ is scale-dependent, but our results imply that it is. Comparing *K*_att_ and *K*_det_ at different scales, we observed that in the column tests, *K*_att_ was about 2 orders of magnitude higher than in the field, while *K*_det_ was less than 1 order of magnitude higher in the field than in the column tests. This indicates that both *K*_att_ and *K*_det_ are scale dependent, albeit *K*_det_ to a lesser extent; the high standard deviations for *K*_det_ should be noted.

As with the *K*_att_/*K*_det_ ratio, it has been argued that α-values might be similar between column and field scales, for short travel distances.^68^ This was not found to be the case for this study, which has a travel distance in the field of 25 m, as α was lower by around 2-log in the field. This implies that α is scale-dependent; however, the material at our field site is poorly sorted (*C*_U_ = 38) and this might influence the results, as CFT was developed for uniform soils.^40^ Therefore, α-values might be similar between the column and field scale for other, more uniform porous media. Furthermore, Vienna tap water is more oxic than the groundwater (Table 1). This might lead to precipitation of certain oxides, which would increase attachment.^68,69^

The results show that even though the same material was used, there was more removal and attachment (λ, *K*_att_, α) in the columns compared to the field, regardless of which modeling method was used. This upscaling phenomenon of relatively more colloidal removal per distance at the column scale compared to the field is well established, but there is no generally accepted reason for this. It has been hypothesized that it may be due to the smallest representative elementary volume (REV) (Pang, 2009), which could capture heterogeneities, and is therefore dependent on type of aquifer material. In order to explore this phenomenon in more detail, the values of λ in the column and field were compared to other studies that were also performed at both scales in the same porous media. Since it was shown that tailing, attachment, and detachment were important in our tests, it would have been better to compare the ratio of attachment and detachment rates, *K*_att_ and *K*_det_, to the literature. Unfortunately, not many studies that were done in the field and use the same porous media in the column, include attachment/detachment modeling and, for this reason, we have chosen to compare the ratio of λ, which was available.

**Figure 5:**
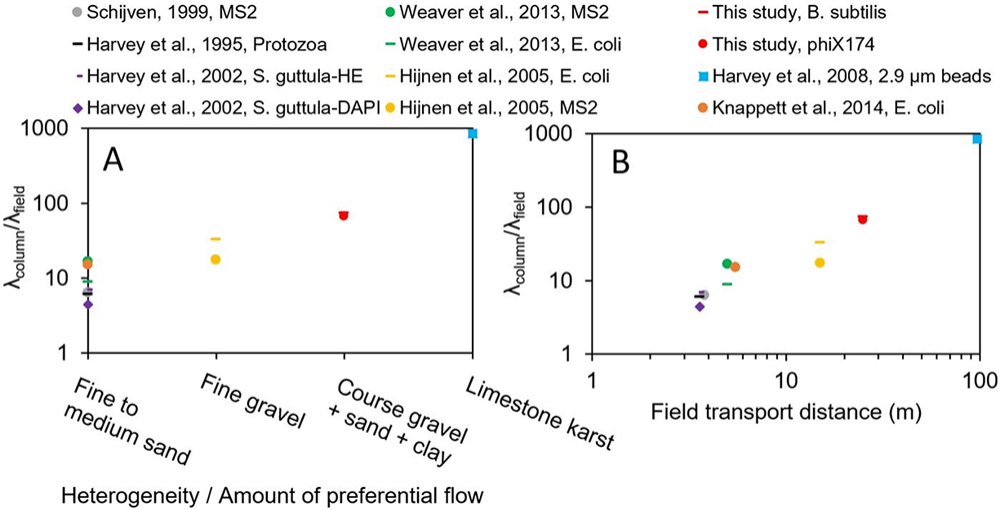
(A) Calculated ratios of λ_column_/λ_field_ (according to eq 4) from published studies done in columns and field tracer tests in materials of varying heterogeneity and/or varying amounts of preferential flow. (B) The same ratios of λ_column_/λ_field_ plotted against the transport distance in the field. Values for λ (log m^–1^) taken from Pang.^17^

The ratio of removal rates λ, in the column to λ, in the field, or in other words, λ_column_/λ_field_, is around 100. Table S2, in the SI, shows studies done by others that have been carried out with microorganisms on both the column and field scale, mostly using the same material at both scales. The literature seems to indicate that the ratio of λ_column_/λ_field_ is mostly dependent on the heterogeneity of the subsurface (Figure 5A). Notably, this λ-ratio is never 1 (which means that removal in the column is always significantly higher than in the field), even in completely homogeneous material.^23^ This suggests that there is something inherent about the way column and field tests are performed that results in a higher λ_column_. Smith et al. (1985) showed that a disturbed column had 3-log higher removal of *E. coli* than an undisturbed column of the same size, which indicates that macropore destruction has a strong effect on removal rates.^70^ Another difference between the column and the field is the arrangement of grains. Grains are not perfect spheres and are deposited horizontally in nature, in line with the flow direction through the aquifer. During column tests, this flow is vertical and perpendicular to this layering effect, which might influence removal during transport. Lastly, we are comparing columns on the centimeter-scale to flow paths on the meter-scale in the field. Along these flowpaths there may be cracks or lenses of higher permeability that are larger than the column size itself. Therefore, extrapolation from the column to the field scale is problematic because we inherently assume a field site without these elements of preferential flow.

Figure 5B addresses the issue of transport distance (*x*-axis), which is usually chosen depending on the material type (Figure 5A), to allow for preferential flow paths. This is an attempt at solving the upscaling problem with the use of characteristic lengths, similar to REV, as mentioned previously. It has been suggested that using characteristic lengths is a good way to parametrize the order-of-magnitude of problems, which might lead to enhanced insight into processes at different scales.^71^ In the studies considered, sand aquifers have a λ_column_/λ_field_ ratio of around 10 (Figure 5A). Well sorted gravels have a slightly higher ratio,^23,72^ while poorly sorted gravels (this study) have a ratio of around 100. Karstic aquifers (or other aquifers with extreme preferential flow) can have a λ-ratio of 1000.^13^

The destruction of preferential flowpaths due to disturbing the soil when making columns is often cited as a major cause of this, because this leads to all matrix flow in the column, and therefore to higher removal rates.^70,73^ Additionally, removal might happen predominantly in the first centimeters after injection, which leads to a decreasing removal rate (λ) with distance.^74^ This can have multiple explanations, summarized by Pang as being due to straining and heterogeneous/unfavorable attachment.^17^ A popular theory is that favorable attachment sites are progressively “filled up” and blocked, so that the colloids have to travel further to find an attachment site, causing attachment to be nonlinear.^75^ Additionally, colloidal population heterogeneity might lead to viruses or bacteria with higher sticking efficiencies attaching first, and others later or not at all. This theory of fast versus slow attachment would indicate that the slower attaching colloids in the population would be a minority, since most attachment happens right after injection, and Schijven et al. found that the chemical heterogeneity of the aquifer material was more important than the heterogeneity of the colloidal population.^76^ It is also possible that microorganisms attach to other particles like clay and are cotransported, thereby enhancing their travel distance.^68^ Lastly, in sub- or anoxic aquifers with iron-rich groundwater, like the one in this study, iron-oxides might precipitate around the injection well when oxygen is introduced during injection.^77^ This might influence removal rates, because iron oxide grain coatings provide sites for enhanced attachment.^78,79^ To minimize oxidation, a piezometer was used that was never used before as an injection well, only groundwater that was recently pumped and mixed with tracers was reinjected, and injection was always below the water table.

An explanation for the λ-ratio increasing as heterogeneity increases could be that flow through preferential flow paths is faster than fine matrix flow, decreasing attachment, and these preferential flow paths cannot be recreated in the columns. Flow through crack networks can be up to 4 times higher than that of the adjacent matrix.^22,80^ Other authors have made observations about scaling between field tests, noting the inverse relationship between the length of the flowpath and the removal rate λ.^40^ Longer field flow paths have reduced λ, which increases the λ_column_/λ_field_ ratio. The length of the field flow paths considered in the present literature comparison range from 3.6 to 97 m (Figure 5B). This could lead to a bias in the comparison of the λ-ratios in Figure 5A. The flow path lengths of these field studies were probably chosen so that it could capture flow elements typical of the aquifer, i.e., crack networks or lenses of course material. Therefore, even though some bias might exist due to transport distance, heterogeneity of the material may be one of the more important parameters affecting the λ_column_/λ_field_ ratio based on the literature comparison in Figure 5, and transport distance is usually chosen based on heterogeneity. This upscaling ratio may also be altered depending on type of colloid, as well as differences in ionic strength of the groundwater matrix,^45,55,59^ groundwater chemistry, chemical composition of aquifer media, and heterogeneity within the microorganism community, to name a few, but in our study these influences were minimal since the same porous media and colloids were used in the column and the field tests.

Upscaling colloidal transport from the column to the field scale is challenging because of the complex structures often inherent to porous media. These structures create intricate flow patterns which are difficult to quantify, and therefore problematic when upscaling transport processes. In this study, results reveal that column tests overestimate log-removal rates by approximately 2-log in poorly sorted gravel material. Similarly, values for *K*_att_ and the CFT parameter α were overestimated by 1 to 2 log in the column. Preferential flow due to material heterogeneity may be the main driver for this phenomenon, as it is difficult to recreate preferential flow paths in a small column. This needs to be confirmed with more opportunity for comparison with upscaling studies in other types of aquifer materials. Furthermore, we showed that in gravel material, phage phiX174 (as a surrogate for viruses in general) has a slightly higher removal rate compared to spores of *B. subtilis*, possibly because larger colloids (such as *B. subtilis*) are transported more than smaller colloids through preferential flow paths, created by course gravel lenses, and a certain percentage of virus-sized particles are trapped in narrow, dead end pathways.

The environmental implication of this study is that based on the comparison of our study with literature data, preliminary conclusions surmise that the type of porous media affects the upscaling relationship. With this relationship, environmental pollution could be more accurately estimated. However, it is not precluded that other important drivers play an important role, such as the type of microorganism or physicochemical conditions in the subsurface. Future research is planned to test the findings of this study, focusing on mesoscale pathogen transport in a large, undisturbed gravel column.

## Supporting Information Available

Field site material, modeling equations, and literature comparison (PDF)
