# Supplementary material for: Upscaling Transport of Bacillus subtilis Endospores and Coliphage phiX174 in Heterogeneous Porous Media from the Column to the Field Scale
Source: Environ Sci Technol. 2021 Jul 28;55(16):11060–9. doi: 10.1021/acs.est.1c01892 (PMC8375017; doi:10.1021/acs.est.1c01892)
Supplement: Supplementary file 1 — es1c01892_si_001.pdf [file es1c01892_si_001.pdf]

Supporting Information on

"Upscaling transport of Bacillus subtilis endospores and coliphage phiX174 in heterogeneous porous media from the column to the field scale"

Authors: Thomas J. Oudega[1,3,6], Gerhard Lindner[1,4,6], Julia Derx[1,3,6], Andreas H. Farnleitner[2,5,6], Regina Sommer[4,6], Alfred P. Blaschke[1,3,6], Margaret E. Stevenson[1,3,6]

1. Institute of Hydraulic Engineering and Water Resources Management E222/2, TU Wien, Karlsplatz 13, A-1040 Vienna, Austria
2. Research Group Environmental Microbiology and Molecular Diagnostics 166/5/3, Institute of Chemical, Environmental and Bioscience Engineering, TU Wien, Gumpendorferstraße 1a, A-1060 Vienna, Austria
3. Center for Water Resource Systems E222, TU Wien, Karlsplatz 13, A-1040 Vienna, Austria
4. Medical University of Vienna, Institute for Hygiene and Applied Immunology, Water Hygiene, Kinderspitalgasse 15, A-1090 Vienna, Austria
5. Karl Landsteiner University for Health Sciences, Department Physiology, Pharmacology and Microbiology, Research Division Water Quality & Health, Krems, Austria
6. Interuniversity Cooperation Centre (ICC) Water & Health, www.waterandhealth.at, Austria

This Supporting Information consists of:

- 5 Pages (S1-S5)
- 1 Figure (Fig. S1, page S2)
- 3 Equations (Eq. S1-3, page S3)
- 1 Table (Table S1, page S4)
- Bibliography (Page S5)

Fig. S1. Field site material. These pictures were taken immediately after removing the material from the field site at the location of P24.

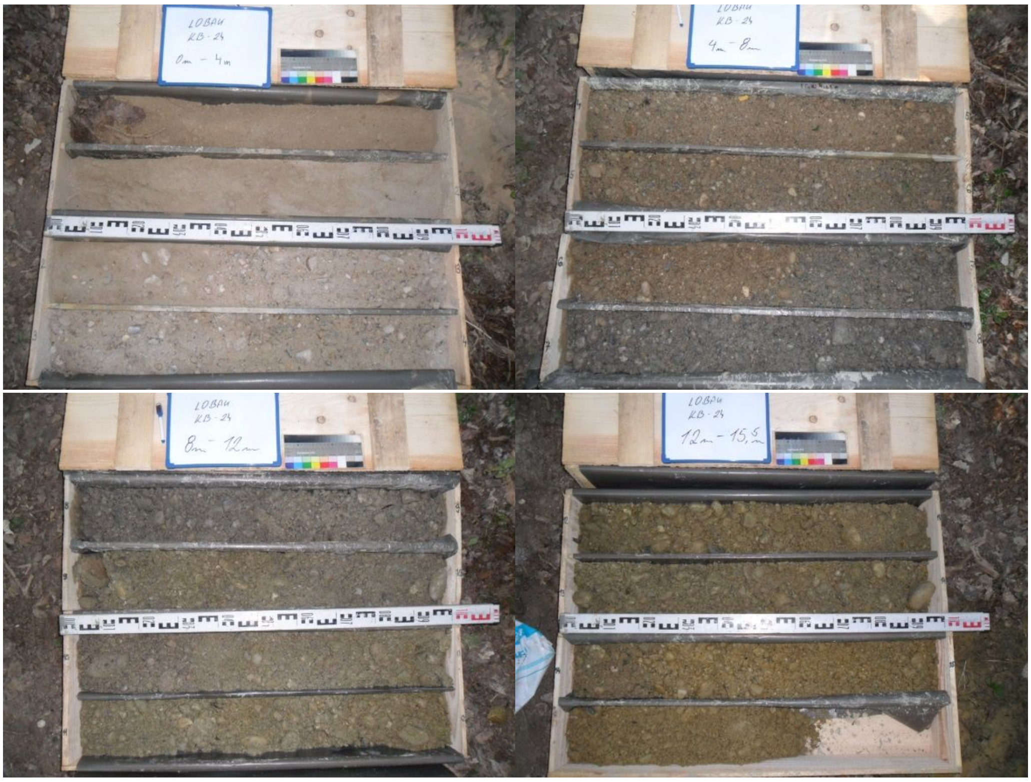

Table S1. Aquifer geochemical properties, material for measurement taken from a borehole at 2 km distance of this study's field site (modified from Peresson, 2014, Table 1).

Depth (m)	4.6	8.0	12.0	13.8	16.0
SiO_2 (%)	58.0	63.0	80.5	71.6	86.0
TiO_2 (%)	0.4	0.1	0.2	0.4	0.3
Al_2O_3 (%)	7.8	2.9	4.2	9.9	4.9
FeO (%)	2.2	0.9	1.1	2.4	1.1
MnO (%)	0.05	0.02	0.02	0.03	0.03
MgO (%)	4.8	5.2	1.9	1.8	0.7
CaO (%)	11.9	12.2	5.8	5.3	2.5
Na_2O (%)	0.9	0.3	0.33	0.50	0.4
K_2O (%)	1.4	0.84	1.11	2.04	1.27
P_2O_5 (%)	0.07	<0.01	<0.01	0.01	<0.01
$H_2O^{110° C}$ (%)	0.2	0.1	0.1	0.2	0.2
H_2O+ (%)	0.6	2.4	0.1	1.5	0.4
CO_2 (%)	11.5	11.7	4.6	3.7	1.7
SO_3 (%)	0.03	0.03	0.02	0.03	0.02
**Sum**	**99.8**	**99.7**	**99.9**	**99.5**	**99.5**
As (ppm)	4	1	1	4	1
Ba (ppm)	221	138	173	272	169
Cd (ppm)	<1	<1	<1	<1	<1
Ce (ppm)	27	<3	26	22	23
Co (ppm)	10	5	<5	10	7
Cr (ppm)	43	18	30	59	36
Cs (ppm)	3	<1.5	<1.5	<1.5	3
Cu (ppm)	13	9	11	17	11
La (ppm)	17	<2	4	11	13
Nb (ppm)	8	3	4	8	6
Nd (ppm)	<5	<5	<5	<5	<5
Ni (ppm)	21	9	16	35	16
Pb (ppm)	16	7	8	11	8
Rb (ppm)	48	26	34	66	37
Sr (ppm)	161	115	82	105	68
V (ppm)	48	11	18	59	23
Y (ppm)	18	8	10	15	15
Zn (ppm)	39	17	20	43	24
Zr (ppm)	166	78	96	126	200

Sum (ppm)	862	447	534	864	662
Ttl. sum (%)	99.9	99.7	99.9	99.5	99.6

Modelling equations. The following equations were used for modelling of the column tests and field tests (Bradford et al., 2004; Yao et al., 1971).

$$\frac{\partial C}{\partial t} = D_x \frac{\partial^2 C}{\partial x^2} + D_y \frac{\partial^2 C}{\partial y^2} + D_z \frac{\partial^2 C}{\partial z^2} - v \frac{\partial C}{\partial x} - \frac{\rho_b}{\theta} \frac{\delta S}{\delta t} \qquad \text{(S1)}$$

$$\frac{\rho_b}{\theta} \frac{\delta S}{\delta t} = k_{att} c - \frac{\rho_b}{\theta} k_{det} S \qquad \text{(S2)}$$

where C is equal to the concentration of free microorganisms [L^{-3}], D is spatial dispersion [L^2 T^{-1}], ρ_b is bulk density [M L^{-3}], θ is porosity [-] and S is the concentration of attached particles [M L^{-3}]. Values for k_{att} and k_{det} (attachment and detachment rates [T^{-1}], respectively) were found by calibrating the model to the microbial breakthrough curve (BTC).

The second modeling approach used, for comparison, was colloid filtration theory, or in short CFT (Bradford et al., 2004; Yao et al., 1971) in which k_{att} is described using an attachment efficiency factor (α) and single collector efficiency (η):

$$k_{att} = \frac{3(1-\theta)}{2 \quad d} \alpha \eta v \qquad \text{(S3)}$$

where v is equal to Darcy velocity [L T^{-1}], θ is effective porosity [-], and d is mean grain size diameter [L]. In CFT, η describes the amount of particles colliding with the collector, which is calculated using equations from Tufenkji & Elimelech (2004), while α describes how many of the collided particles are successfully attached to the collector. Detachment rate values found

during attachment/detachment modeling were kept constant during modeling with CFT. In these modeling runs, the value for α was found by calibration to the microbial BTC.

Table S1. Literature values for the comparison of λ (log/m) on the field and column. Comparisons 1-6 are taken from Pang (2009). The parameter x is the length of the flow path in the test (m).

Comparison	Author	Year	Site	Microbe	Media	Log_{10} reduction	x (m)	λ (log/m)	$\lambda_{column}/\lambda_{field}$
1a	Medema et al.	2002	Field	*E. coli*	Coarse and fine gravel with sand	4	15	0.27	32.96
	Hijnen et al.	2005	Column	*E. coli*		4.1 - 4.8	0.5	8.2 - 9.6	
1b	Medema et al.	2002	Field	F-RNA Phages	Coarse and fine gravel with sand	4	15	0.27	17.41
	Hijnen et al.	2005	Column	MS2 phages		1.3 - 3.4	0.5	2.6 - 6.8	
2	Schijven	1999	Field	MS2 phages	Dune sand (Castricum)	3.3	3.8	0.87	6.32
	Hijnen et al.	2005	Column	MS2 phages		2.2 - 3.3	0.5	4.4 - 6.6	
3	Harvey et al.	1995	Field	Protozoa	Sand and fine gravel (Cape Cod)	2.92	3.6	0.81	6.01
			Column	Protozoa		2.92	0.6	4.87	
4	Harvey et al.	2008	Field	2.9 μm microspheres	Karst limestone	1.4	97	0.01	847
			Column	2.9 μm microspheres		1.4	0.17	8.47	
5a	Harvey et al.	2002	Field	Spumella guttula-DAPI	Cape Cod - Sandy Gravel	0.39 - 0.72	1 - 3.6	0.18 - 0.39	4.40
			Column	Spumella guttula-DAPI	0.5-1 mm sand (Cape Cod)	0.41 - 1.1	0.6	0.68 - 1.83	
5b			Field	Spumella guttula-HE	Cape Cod - Sandy Gravel	0.89 - 2.0	1 - 3.6	0.56 - 0.89	6.90
			Column	Spumella guttula-HE	0.5-1 mm sand (Cape Cod)	3	0.6	5	
6a	Weaver et al.	2013	Field	*E. coli*	Medium to coarse sands		1 - 5	0.53	8.85
			Column	*E. coli*	Clean silica sands (Grade F-50)		0.5	4.69	
6b			Field	MS2 phages	Medium to coarse sands		1.5	0.36	16.67
			Column	MS2 phages	Clean silica sands (Grade F-50)		0.5	6	

7	Knappett et al.	2014	Field	*E. coli*	Sand (Char Para)		2.5 - 7	1.0		15
			Column	*E. coli*			0.1	15		
8a	This study	2020	Field	phiX174	Alluvial sandy gravel (Lobau)	4.46	25	0.15		66.78
			Column	phiX174		4.96	0.5	9.93		
8b			Field	*B. subtilis* spores		2.88	25	0.12		73.20
			Column	*B. subtilis* spores			4.21		0.5	8.43

References for the Supporting Information

Bradford, S. a, Bettahar, M., Simunek, J., van Genuchten, M.T., 2004. Straining and attachment of colloids in physically heterogeneous porous media. Vadose Zo. J. 3, 384–394. https://doi.org/10.2136/vzj2004.0384

Tufenkji, N., Elimelech, M., 2004. Correlation Equation for Predicting Single-Collector Efficiency in Physicochemical Filtration in Saturated Porous Media. Environ. Sci. Technol. 38, 529–536. https://doi.org/10.1021/es034049r

Yao, K., Habibian, M.T., O'Melia, C.R., 1971. Water and Waste Water Filtration: Concepts and Applications. Environ. Sci. Technol. 5, 1105–1112. https://doi.org/10.1021/es60058a005
